# Plant-Derived Vesicle-like Nanoparticles: The Next-Generation Drug Delivery Nanoplatforms

**DOI:** 10.3390/pharmaceutics16050588

**Published:** 2024-04-26

**Authors:** Xiaoxia Wang, Congling Xin, Yu Zhou, Tao Sun

**Affiliations:** 1 Key Laboratory of Smart Drug Delivery (Ministry of Education), Minhang Hospital, State Key Laboratory of Medical Neurobiology and MOE Frontiers Center for Brain Science, Department of Pharmaceutics, School of Pharmacy, Fudan University, Shanghai 201203, China; 22301030052@m.fudan.edu.cn; 2 Department of Gynecology, Fudan University Shanghai Cancer Center, Minhang District, Shanghai 200240, China; 3 Department of Interventional Radiolagy, Ruijin Hospital, School of Medicine, Shanghai Jiao Tong University, Shanghai 200025, China; zy01c36@rjh.com.cn

**Keywords:** nanotherapeutics, plant-derived vesicle-like nanoparticles, drug delivery systems

## Abstract

A wide variety of natural bioactive compounds derived from plants have demonstrated significant clinical relevance in the treatment of various diseases such as cancer, chronic disease, and inflammation. An increasing number of studies have surfaced that give credence to the potential of plant-derived vesicle-like nanoparticles (PDVLNs) as compelling candidates for a drug delivery system (DDS). PDVLNs are cost-effective production, non-toxicity and non-immunogenicity and fascinating bi-ocompatibility. In this review, we attempt to comprehensively review and consolidate the position of PDVLNs as next-generation drug delivery nanoplatforms. We aim to give a quick glance to readers of the current developments of PDVLNs, including their biogenesis, characteristic features, composition, administration routes, advantages, and application. Further, we discuss the advantages and limitations of PDVLNs. We expect that the role of PDVLNs in drug delivery will be significantly enhanced, thus positioning them as the next generation of therapeutic modalities in the foreseeable future.

## 1. Introduction

A drug delivery system (DDS) serves as a carrier to deliver the active pharmaceutical ingredient, which is essential for eliciting the desired therapeutic response. Without such a system, the nonspecific drug accumulation of a drug could lead to toxicity, thus potentially nullifying the therapeutic process [1]. When developing a novel DDS, it is crucial to evaluate its effectiveness across multiple dimensions. Despite decades of intensive research into nanoparticle delivery systems, the progression of these technologies into clinical applications has been hindered by inherent limitations, with only a few systems successfully reaching clinical use.

Hence, scientists have tried to shed light on exosomes for their role as important mediators of intercellular communication through the transfer of active biomolecules, anticipating that they may provide useful developments. Exosomes are vesicles released by cells into extracellular space [2]. Historically, research has predominantly focused on mammalian exosomes, which have, disappointingly, exhibited several problems. In fact, the clinical application of mammalian exosomes is hindered in many ways, including limited yield [3], rapid blood clearance [4], and cancer-stimulating risk [5]. 

Fortunately, an increasing number of studies have demonstrated that the biochemical composition of plant-derived vesicle-like nanoparticles (PDVLNs) overlaps with those previously identified for mammalian exosomes [6], thus encouraging researchers to explore the intercellular communication role of PDVLNs and to look for developmental space in drug delivery [7]. PDVLNs are one kind of exosomes, and they are also vesicles that are released by plant cells. It should be added that there are many terms used, including “Plant-derived extracellular vesicles”, “Plant exosome nanovesicles”, and “Plant-derived nanovesicles” [8,9,10]. In this review, we will refer to these vesicles as plant-derived vesicle-like nanoparticles (PDVLNs). 

Living up to our expectation, PDVLNs offers significant advantages over synthetic carriers and mammalian exosomes with their non-toxic, low immunogenic, good biocompatible, and high yield properties, which are as such due to their widely available and natural source. These exceptional biological properties allow PDVLNs to compensate for the high cost, safety, and even religious and ethical issues that mammalian exosomes may bring without compromising the capacity of drug loading. Due to the natural biochemicals of the original plant, plant-derived vesicle-like nanoparticles (PDVLNs) have a pronounced favorable therapeutic effect, which has been demonstrated in numerous disease models. Moreover, a fair portion of them have already been involved in developing novel drugs [11,12,13].

**Figure 1:**
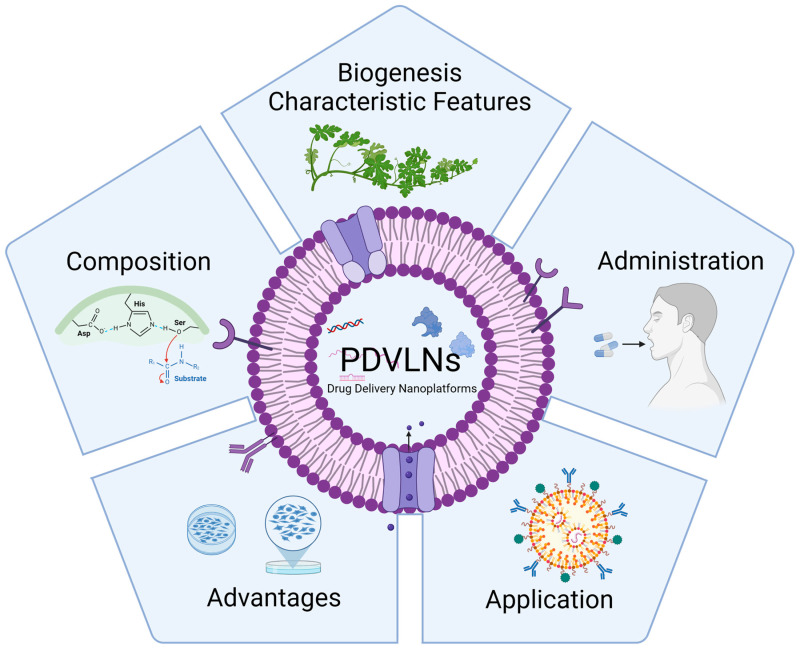
In the following sections, we will review PDVLNs by focusing on their biogenesis, characteristic features, composition, administration routes, advantages, and application. Created with biorender.com, access on 12 April 2024.

But, unfortunately, original research articles in this field are scarce, thus leading to limited numbers of PDVLN-related reviews, especially in terms of drug delivery systems [14]. In this review, we attempted to comprehensively review the possibility of a promising drug delivery platform being created for PDVLNs due to the different facets they possess based on their intrinsic biological characteristics (Figure 1).

## 2. The Biogenesis and Characteristic Features of PDVLNs

In the early 1960s, multivesicular bodies (MVBs) were observed fusing with plasma membranes, which then led to the release of exosome-like vesicles into the extracellular space in carrot cell cultures [15]. Since then, while our understanding of PDVLN biogenesis has evolved and many studies have successfully isolated and characterized PDVLNs, the exact mechanism of its biogenesis remains ambiguous and controversial.

There are generally believed to be at least three biogenesis pathways: (1) the MVB pathway; (2) the exocyst–positive organelle (EXPO) pathway; and (3) the vacuolar pathway [2]. Of these, the MVB pathway is the most commonly reported [2]. In this pathway, the inward budding of the endosomal membrane forms MVBs, and the fusion of MVBs with plasma membranes (PMs) results in the release of exosome-like vesicles. The ESCRT complexes play a crucial role in the cargo sorting within PDVLNs. For example, An et al. reported the proliferation and fusion of MVBs with plasma membranes (PMs) in Arabidopsis leaf tissue, which suggests that plants produce intraluminal vesicles through the MVB pathway in response to various environmental stresses [16]. 

Compared with the MVB pathway, the EXPO pathway and vacuolar pathway are forms of unconventional secretion. In the EXPO pathway, EXPOs, rather than MVBs, fuse with PMs and release its internal vesicles into the cell wall. Different from MVBs, EXPOs are independent of the endocytic pathway and are unaffected by inhibitors of the secretory and endocytic pathways [17,18]. Additionally, the central vacuole in Arabidopsis has been reported to externally release antibacterial proteins by fusing with plasma membranes [8]. 

PDVLNs are natural nanoparticles secreted by edible plants including grape [19], grapefruit [20], ginger [21], bitter lemon [22], broccoli [23], carrot [15,24], apples [25], orange [26], Arabidopsis [27], etc.

There are various isolation methods of PDVLNs, such as size-exclusion chromatography (SEC), ultrafiltration, polymer-based precipitation ultracentrifugation, precipitation, and immunoaffinity capture [28]. Among these methods, ultracentrifugation is the most commonly used isolation method, and more than half of the respondents use a combination of methods in a survey of practices for the isolation of PDVLNs [29]. Although the isolation methods of PDVLNs are similar with those of mammalian exosomes, the detailed steps of PDVLNs tend to be less complex. For example, when isolating and producing MSC-derived exosomes (MSC-Exos) by ultracentrifugation, the cell culture environment, cultivation system, dissipation enzyme, and culture medium must be strictly controlled, and significant centrifugal forces up to 1,000,000× *g* are required to separate MSC-Exos [30]. In comparison, after grinding the plant tissue and collecting filtered fluid, PDVLNs can commonly be produced by centrifuging at a comparatively low speed [31].

The diameters of PDVLNs range from 50 to 500 nm, and they are mainly dependent on the source plant. Their appropriate size satisfies the premise of using them as drug delivery nanoplatforms. The size distribution of PDVLNs from various plant sources and different isolation methods is summarized below (Table 1).

## 3. Composition of PDVLNs

**Figure 2:**
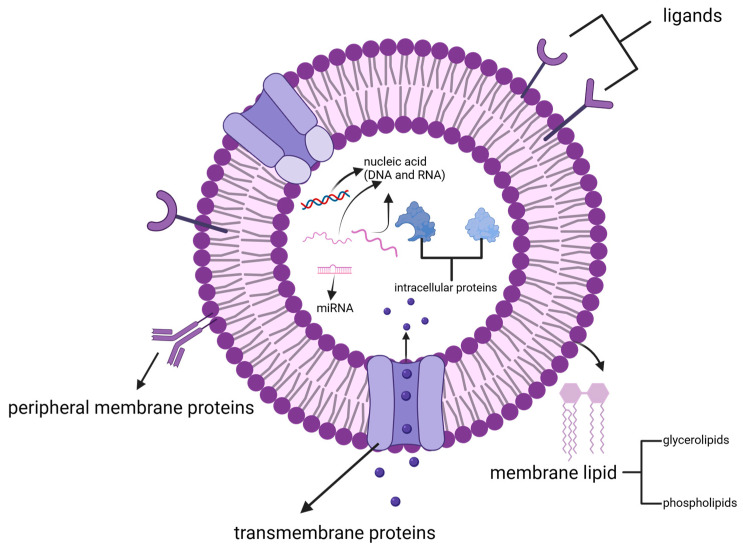
PDVLNs are mainly composed of bioactive lipids, proteins, RNA, and nucleic acids. Created with biorender.com, accessed on 9 April 2024.

PDVLNs are replete with bioactive lipids, proteins, RNA, and nucleic acids [38] (Figure 2), all of which perform a diverse array of activities on physiological and pathological processes [39]. Meanwhile, the composition of PDVLNs is crucial for their biochemical characterization. With respect to analyzing their biochemical composition, PDVLNs and mammalian exosomes have been investigated via several mutual and common techniques, including enzyme-linked immunosorbent assay (ELISA), sodium dodecyl sulfate–polyacrylamide gel electrophoresis (SDS–PAGE), Western blot (WB), and liquid chromatography–tandem mass spectrometry [4,5,40,41]. In this section, we will detail the composition of PDVLNs, focusing on lipids, proteins, and nucleic acids (Table 2).

### 3.1. Lipids

Lipids are an important kind of biomolecules that play a key role in cellular response; they maintain the exosome’s structural stability, as well as promote the uptake and retention of bioactive cargos and intercellular communication. Lipids are the fundamental components of the membrane structures of PDVLNs. The total lipid profiling of PDVLNs has revealed that lipids can be divided into two distinct groups: glycerolipids and phospholipids [42]. Specially, PDVLNs accumulate diverse lipid content such as phosphatidic acid (PA), phosphatidylethanolamine (PE), phosphatidylcholine (PC), phosphatidylinositol (PI), digalactosyl diglyceride (DGDG), and monogalactosyldiacylglycerol (MGDG), which endow them with regulatory activities [43].

A growing body of research has consistently demonstrated that the lipid compositions of PDVLNs varies according to the plant species. For example, while orange-derived PDVLNs contain about 40% PE, 25% PC, 12% PI, and 5% PA [5], turmeric-derived PDVLNs are enriched with about 42% DGDG, 12% MGDG, 15% PA, and 5% PC [44]. The lipid profiles of orange-derived PDVLNs are nearly identical to those of grapefruit [5], which means ginger-derived PDVLNs are rich in PA, and PC is the main ingredient in the lipids of grapefruit-derived PDVLNs.

These phospholipids mentioned are essential for the function of PDVLNs. For example, PA can influence the uptake of ginger-derived PDVLNs by Porphyromonas gingivalis and, in turn, the therapeutic effect of ginger-derived PDVLNs for chronic periodontitis [45]. PC is a source of choline in the body and is known to be involved in the various life activities of organisms through membrane-mediated cell signaling [46].

Interestingly, the different lipid profiling of PDVLNs leads to their different distribution [47]. PDVLNs rich in PA, like those from ginger, are preferentially taken up by Lactobacillus rhamnosus, whereas those rich in PC, like from grapefruit, are favored by intestinal Ruminococcaceae [48].

Despite their apparent roles in the composition and uptake of PDVLNs, our understanding of lipids in PDVLNs is still primitive; thus, further study of the lipids in PDVLNs, especially lipidomic analysis on PDVLNs from different sources, is necessary.

### 3.2. Proteins

In general, the protein content in PDVLNs is significantly lower than that in mammalian exosomes [49]. For example, PDVLNs, such as those derived from ginger, typically contain only 28 proteins, whereas mammalian exosomes can contain more than 1000 proteins. The proteins found in PDVLNs are crucial to their functionality, exhibiting high specificity depending on the plant species from which they are derived. For example, unlike ginger-derived PDVLNs, it has been reported that 1438 proteins have been found in the *Arabidopsis* leaf-derived PDVLNs [50].

PDVLN proteins can be categorized primarily into peripheral membrane proteins, transmembrane proteins, and intracellular proteins [14]. In fact, a majority of PDVLN proteins are intracellular [51]. These intracellular proteins include actin and proteolytic enzymes (like lipoxygenase, glutamine synthetase, glucose-6-phosphate isomerase, and CTP synthase [51]). And, as is the case for a few membrane proteins, some of them have been reported to be ATPases, aquaporins, chloride channels, ATP binding cassette (ABC) transporters, and tetraspanins [52]. 

Of note, these proteins are integral to intercellular communication and cellular functions. Several studies have reported that the cytosolic proteins in PDVLNs include the enzymes involved in cell wall remodeling and that they provide antifungal and antibacterial defenses against invading pathogens [2].

Although extensive research has detailed the protein composition of PDVLNs and their diverse functions, the biggest difficulty—establishing a complete protein database of PDVLNs—is still difficult as the protein databases of PDVLNs change depending on the conditions. Therefore, further proteomic analysis, as recommended by the International Society for Extracellular Vesicles, is crucial for advancing PDVLN research [53].

### 3.3. Nucleic Acids

Many studies have verified that PDVLNs are rich in nucleic acids, predominantly RNAs, with only a few instances of DNA reported. These RNAs include messenger RNA (mRNA), microRNAs (miRNAs), small regulatory RNAs (sRNAs), and long noncoding RNAs (lncRNAs). These RNAs can play a role in intercellular and even interspecies communication [54,55]. 

Thanks to the lipid bilayer structure of PDVLNs, miRNAs, which are unstable when naked, circumvent their early obliteration by RNases [56]. On the other hand, miRNAs can be found in various bodily fluids through passive leakage and the active secretion of membrane vesicles such as exosomes or the protein–miRNA complex [42]. These miRNAs inhibit the translation of targeted mRNAs and are involved in a variety of physiological and pathological processes [57,58,59]. 

Substantial research supports these findings [37,60,61,62]. For instance, Xiao et al. identified a variable number of miRNA types across 11 different plant species in PDVLNs, thereby indicating that high miRNA expression may relate to inflammatory responses and cancer-related signaling pathways [12]. Moreover, recent studies have demonstrated that the miRNAs in PDVLNs can alter the gut microbiome in patients in subjects with colorectal adenoma through the interactions of metabolites with the *taxa Lactobacillus rhamnosus* [63]. Therefore, miRNAs in PDVLNs may emerge as a new class of cross-kingdom modulators at the molecular level, particularly in terms of mediating animal–plant interactions [28]. 

The unique capability of PDVLNs to transport various RNAs highlights their potential as drug delivery platforms. But, when confronted with obvious research gaps such as their influence on the uptake of recipient cells, the deeper study of the types and functions of the nucleic acids of PDVLNs, mainly microRNAs, will be of considerable significance.

**Table 2:** Different sources of PDVLNs, their composition, and therapeutic activity.

Source	Lipid	Protein	Nucleic Acids	Therapeutic Activity	Ref.
Orange	PE (~40%) PC (~25%) PI (~12%) PA (~5%)	/	miRNA	Anti-obesity Anti-inflammatory	[5]
Turmeric	DGDG (42%) MGDG (12%) PA (15%) PC (5%)	/	miRNA	Anti-inflammatory (colitis)	[44]
Ginger	PA (37.03%) DGDG (39.93%) MGDG (16.92%)	/	miRNA	Anti-liver damage	[64]
Grapefruit	PE (45.52%) PC (28.53%)	Actin Beta-tubulin Capsid protein Chalcone synthase	miRNA	Anti-inflammatory (bowel)	[65]
Cabbage	/	Actin [Brassica oleracea] Ribosomal protein	miRNA	Anti-inflammatory (colitis)	[66]
Bitter lemon	/	Heat shock protein 70 *S*-adenosyl-homocysteinase Glyceraldehyde 3 phosphate dehydrogenase	miRNA	Anticancer (oral squamous cell carcinoma)	[67]
Strawberry	/	/	miRNA (miR168b-5p miR396a-5p miR159b-3p miRNA159a)	Antioxidant	[32]

## 4. The Administration Routes of PDVLNs

In the advancement of drug delivery nanoplatforms, enhancing the effectiveness of medications holds utmost significance, which necessitates a discussion on administration routes. PDVLNs have been delivered into experimental subjects through various routes, including oral administration, intravenous (IV) injection, intranasal administration, and transdermal delivery (Table 3). These successful administration routes have also laid the groundwork for the subsequent development of PDVLNs as effective drug delivery nanoplatforms. In the following sections, we will provide a detailed introduction of these administration routes.

### 4.1. Oral Administration

Oral administration is a highly preferred and convenient method for delivering pharmaceuticals as it offers several advantages, including reduced risk of infection (unlike IV injection), the avoidance of blood clearance, improved patient compliance, and non-invasiveness [68,69]. Unfortunately, many drugs struggle with poor oral bioavailability, thus limiting their suitability for this route. For instance, macrophage-derived exosomes (MDEs) are rarely used in oral applications in various disease models, and they are typically administered via IV injection. Remarkably, PDVLNs demonstrate a unique capability to effectively encapsulate both hydrophilic and hydrophobic drugs, as well as gene therapy agents, thus maintaining stability under the harsh conditions of the gastrointestinal tract in mouse models. Furthermore, PDVLNs can traverse biological barriers to reach target tissues, and they are primarily facilitated by intestinal macrophages and stem cells [66,70].

In one study, orally administered tea-leaf-derived natural nanoparticles (NTs) were found to efficiently curb the inflammation caused by ulcerative colitis in mouse models, restore disrupted colonic barriers, and enhance the diversity of gut microbiota, thereby preventing and treating the inflammatory bowel disease [36]. Therefore, PDVLNs are regarded as highly promising options for the development of oral delivery systems.

Of note are the excellent gastrointestinal tolerance of PDVLNs, which grants them the biggest advantage over MDEs for oral delivery [71]. While most mammalian exosomes are unable to be orally administered for their instability in gastrointestinal conditions, PDVLNs exhibit a high resistance to digestive enzymes, including gastric pepsin, intestinal pancreatin, and bile extract solutions [65,72]. However, their excellent gastrointestinal tolerance has only been hypothesized thus far, and it has also been conjectured that the gastrointestinal tolerance of PDVLNs may result from the ordered plasma membrane structure or lipid composition of PDVLNs [8].

### 4.2. IV Injection

IV injection is commonly used in treatment due its high bioavailability, avoidance of the first-pass effect, and its rapid effect [73]. For example, ginger-derived exosome-like nanovesicles (GDENs) for siRNA delivery through IV administration have been observed to inhibit the tumor growth in a xenograft model, thus highlighting the potential of IV injection as a feasible route of drug administration for PDVLNs [35]. But direct IV injection of PDVLNs is generally not recommended due to safety concerns as the investigation of the composition of PDVLNs remains incomplete, and there may be unknown substances present.

A recent study has also corroborated this. Chen et al. found higher levels of, in comparison to the oral route, alanine aminotransferase (ALT), complement C3, and aspartate aminotransferase (AST) in mice after the natural exosome-like nanovesicles from edible tea flowers (TFEN) were administered intravenously, thereby indicating higher liver toxicity [74]. The higher toxicity is likely due to the strong immune responses caused by the surface contents (proteins, polysaccharides, and lipids) of TFENs after IV injection, whereas these components might be degraded in the gastrointestinal tract when administered orally. In addition, although TFENs accumulated less in the lungs with metastatic breast tumors when administered orally, this route achieved a comparable reduction in mortality rate and an increase in survival rate, which could potentially be linked to effects on the intestinal microbiota. These findings suggest that oral administration may offer more potential for treating gastrointestinal diseases, whereas IV injection could be more effective when surface contents are crucial for therapeutic activity.

### 4.3. Intranasal Administration

Another possible approach route is intranasal administration, which is commonly used for treating pulmonary diseases and offers a direct delivery method to the respiratory system and even the brain [75,76]. Notably, given the challenges posed by the blood–brain barrier (which often hampers the central nervous system drug development), this route presents a promising alternative [77].

Zhuang et al. developed a nanovector hybrid based on grapefruit-derived PDVLNs, and they found that through the intranasal route, this nanovector hybrid could reach the brain within 1.5 h, thus effectively delaying the growth of brain tumors [34]. This study encourages a further exploration of PDVLNs as platforms for delivering biological cargo to the brain via the intranasal route.

### 4.4. Transdermal Delivery

Considering the cargo entrapment efficacy and enhanced skin permeation—given that PDVLNs structurally resemble liposomes—there is potential for administering PDVLNs via transdermal delivery, especially for treating skin melanomas [78,79]. It has been reported that broccoli-derived PDVLNs are highly lipophilic and can be deployed to penetrate deeply into the porcine skin layers. The fusion of these nanovectors with keratinocytes was confirmed through the transport of a fluorescent substance encapsulated within the cells, as well as the presence of broccoli proteins in the plasmalemma of keratinocytes [6].

Interestingly, several studies have compared the biodistribution of PDVLNs via different administration routes. For instance, the intranasal route is frequently employed for brain-targeted drug delivery [31,34,80], whereas PDVLNs tend to accumulate in the liver and lungs when administered intravenously [74]. However, when administered orally, PDVLNs typically appear in the stomach and gut [36].

## 5. Advantages of PDVLNs

The potential of PDVLNs as drugs is attracting widespread attention, with research on grapefruit-derived PDVLNs being the most extensive in terms of their anti-tumor effects. Grapefruit-derived PDVLNs have shown significant efficacy in the field of anti-tumor treatment, with substantial advantages in key drug properties such as preparation methods, yield, cell uptake efficiency, and safety (Table 4). Additionally, PDVLNs from grapes (NCT01668849) and aloe (NCT03493984) are currently undergoing clinical trials as innovative therapeutic options.

These encouraging developments stem from its exceptional biological properties, which are equally important in the development of drug delivery nanoplatforms.

### 5.1. Cost-Effective, Sustainable, and Large-Scale Production

One of the benefits of PDVLNs is that they can be derived from numerous edible plants, which allows for their cost-effective, sustainable, and large-scale production compared to artificially synthetic nanoparticles or mammalian exosomes [81,82]. Compared to animal/human cells, PDVLNs have higher production rates and shorter extraction periods, thus making PDVLNs a promising biological material with significant application potential. In contrast, mammalian exosomes often have lower yields and limited sources such as milk, urine, blood, and bile [83,84]. Therefore, the emergence of PDVLNs provides new possibilities to overcome these limitations.

### 5.2. Editability and Flexibility

PDVLNs break free from the ethical constraints of animal and human research/medicine. With the superior versatility of plants, scientists can more easily edit plant cells and influence the properties of their exosomes. This flexibility allows scientists to explore the potential application value of PDVLNs more freely. In addition, the large-scale production of PDVLNs offers more space for modification and functionalization. The functional proteins and polysaccharides in PDVLNs differ significantly from those in mammalian exosomes, thereby helping to avoid the potential functions inherited from animal sources such as toxicity, immunogenicity, and off-target effects. Scientists can modify PDVLNs through methods like membrane insertion according to their needs to enhance their functionality. This gives us more expectations for its innovative role in drug delivery nanoplatforms.

### 5.3. Non-Toxicity and Non-Immunogenicity

An ideal drug delivery system must satisfy both non-toxicity and non-immunogenicity. PDVLNs exhibit enhanced safety and minimal cytotoxicity because of their natural provenance when compared to synthetic nanoparticles and liposomes [85]. They are sourced from a variety of plants, including lemon [86], shiitake mushroom [87], and Asparagus cochinchinensis [88], with no reported toxicity or inflammation. 

Exosomes sourced from edible fruits and vegetables exhibit lower immunogenicity, thus offering advantages for their use in the field of biomedicine. The low immunogenicity of PDVLNs implies better biocompatibility in medical treatment and biomaterial applications (especially in drug delivery nanoplatforms), thereby helping to reduce potential immune rejection reactions. 

In addition, many factors contribute to low toxicity. Firstly, PDVLNs are mostly derived from edible plants and anticipated to be deemed suitable for human consumption. Second, contrary to mammalian or bacterial cell-derived exosomes [89], plants do not harbor zoonotic or human pathogens, thereby minimizing the possibility of the unintentional transmission of pathogenic genes or proteins.

### 5.4. Fascinating Biocompatibility

The fascinating biocompatibility of PDVLNs is also valuable. Many studies have reported that there is no significant effect on the physiological and biochemical parameters of experimental animals, thus highlighting their safety [90,91,92]. Of note, PDVLNs enjoy the perk of cross biological hindrances such as the blood–brain barrier without eliciting the inflammatory response. Zhuang et al. found that, in mouse models, grapefruit-derived PDVLNs were able to deliver therapeutic microRNA (miRNA) to the brain tumor successfully, thus suggesting the biocompatibility of PDVLNs [34]. In additions, while PDVLNs are capable of traversing various physiological barriers, they do not cross the placental barrier, thus avoiding potential pregnancy complications. In one study, no fluorescent signal was detected in the placenta after pregnant mice were injected with grapefruit-derived PDVLNs, thereby paving the way for the potential use of PDVLNs in pregnant women for delivering drugs in the future [93].

### 5.5. Wide Variety of Sources

Above all, the most intriguing property of PDVLNs are related to their wide variety of sources. According to the Food and Agriculture Organization, there exists a vast array of more than 50,000 therapeutic plants globally. These medicinal plants hold immense potential as valuable natural resources for contemporary pharmaceutical exploration even though the active content of most plants have not yet been identified and evaluated [94]. Some PDVLNs derived from medicinal plants are expected to retain the pharmacological activity of their parent plant. For example, exosomes produced by medicinal plants like ginseng [4], ginger [48], grapes [95], and honeysuckle [96] may carry similar active ingredients as their parent plants, thereby indicating potential pharmacological activity. This opens up new possibilities for utilizing PDVLNs as drug delivery systems or carriers, thus expanding the scope and effectiveness of drug therapy.

In addition to the species, countless factors, including the origins, soil condition, agricultural model, and plant organ, influence the trait, composition, and target effect of PDVLNs. For instance, Logozzi et al. found that PDVLNs obtained from organic agriculture displayed a greater overall antioxidant capacity in comparison to PDVLNs obtained from conventional agriculture [97]. In addition, PDVLNs derived from strawberries had a different size distribution pattern of small RNAs compared to that observed in strawberry juice [32]. Similarly, in ginger, the levels and varieties of miRNA were found to be higher in PDVLNs compared to ginger tissue, whereas the tRNA content was observed to be more abundant in ginger tissue than in PDVLNs [98]. While various sources of PDVLNs allow researchers to select the most appropriate nanovesicles for specific diseases, establishing a comprehensive database of PDVLN traits is challenging and raises several issues. On the one hand, the latent toxicity because of the unknown bioactive constituents of plants cannot be overlooked. On the other hand, standardizing PDVLN production increases the complexity of setting standards for cultivation, isolation, characterization, clinical trials, and manufacturing. Establishing good manufacturing practices (GMP) for PDVLNs is therefore imperative.

**Table 4:** Comparison of the advantages and disadvantages of exosomes from different sources in the field of biological applications.

Source	Advantage	Disadvantage	Ref.
Plant	High yield; Environmentally friendly; Biocompatibility; Excellent gastrointestinal tolerance; Free of human pathogens; Targeting ability.	Potential toxicity High heterogeneity; Lack of standardized GMP.	[81,82,89,90]
Mammalian cell (MSC)	Potential therapeutic efficacy; Superior biocompatibility.	Low productivity; Complex preparation steps; High heterogeneity.	[83,84]
Body fluid (milk)	High production; Non-cytotoxicity; Gastrointestinal tolerance; Superior biocompatibility.	Lack of standardized separation method; High heterogeneity.	[99,100]
Bacteria	Targeting potential; Biocompatibility; Stability in vivo.	Low productivity; Low efficacy; Toxicity; High heterogeneity.	[101,102,103]

## 6. The Application of PDVLNs

Numerous research efforts have concentrated on developing targeted drug delivery systems (DDS) that enable the precise targeting of specific cells, tissues, or organs. The innate ability of plant-derived vesicle-like nanoparticles (PDVLNs) to localize at targeted tissues is particularly appealing to researchers because it minimizes cytotoxicity by avoiding systemic effects and reducing off-target interactions. The natural biodistribution of PDVLNs is influenced by a variety of factors, from the selection of the administration route to the source plant’s intrinsic characteristics.

The process of PDVLNs internalization is crucial for their effectiveness; however, the mechanisms through which they target recipient cells are not yet fully understood and warrant further investigation. It remains unclear whether this targeting occurs through a non-specific process like micropinocytosis or a receptor-dependent pathway. Some researchers speculate that certain surface proteins shared by many PDVLNs may act as a general ligand for cell receptors; yet, identifying specific functions of these molecules and ligands remains a challenge.

For now, a mannose-specific binding protein, II lectin, provides convincing evidence for this speculation. Song et al. found that garlic-derived nanovesicles showed less uptake after removing all the surface proteins of garlic-derived nanovesicles. They also ascribed this to the specific interactions between II lectin and CD98 receptors, which are involved in the internalization of the garlic-derived nanovesicles [104]. CD98 receptors are a type II transmembrane protein, and they have been found to be vital in the endocytosis of the vaccinia virus and the internalization of human β-defensin 3 in epithelial cells [105,106].

There have been numerous applications of PDVLNs as a DDS based on its targeting ability, especially for cancer. The present chemotherapeutic approaches in the fight against cancer are still unable to shake off the high toxicity and poor bioavailability that comes with the process, although great efforts have been made into the development of multiple new drug delivery systems such as cell membrane capsules and multiple generations of liposomes [107]. Fortunately, PDVLNs, owing to their intrinsic targeting features, bring hope for a solution to these problems. 

**Figure 3:**
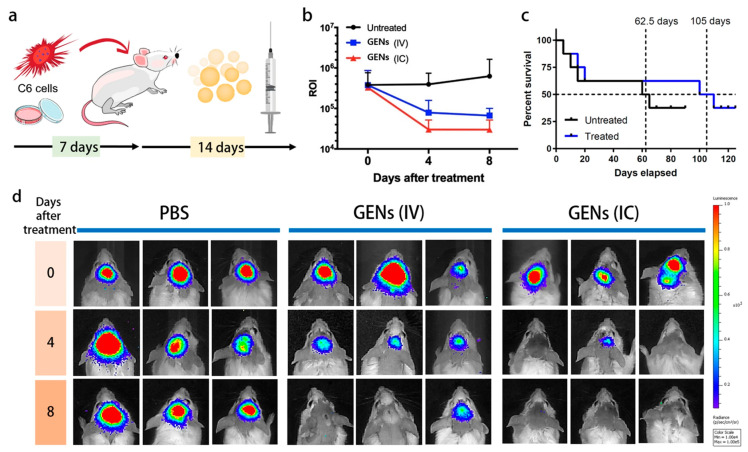
The anticancer effect of ginseng-derived PDVLNs. (**a**) After being implanted with glioma cells into their brains, the mice were given ginseng-derived PDVLNs for 14 days. (**b**) A reduction in ROI was found in the mice treated with ginseng-derived PDVLNs. (**c**) A comparison of the survival rate of mice treated with ginseng-derived PDVLNs and untreated mice. (**d**) A significant reduction in C6 glioma was found in mice treated with ginseng-derived PDVLNs [108].

Take ginseng-derived PDVLNs for example: the composition of ginseng-derived PDVLNs was analyzed, and it was found that ginseng-derived PDVLNs mainly consist of phosphatidylcholines (PC), along with 98 miRNAs and 86 proteins similar to those of *Panax ginseng* [108] (Figure 3). Ginseng-derived PDVLNs have demonstrated high stability, specific targeting abilities, and effective anti-cancer properties in immune regulation; thus, they could be a potential therapeutic option for cancer [109].

In addition, another study found that the improved targeting effect of ginseng-derived PDVLNs, which carry various chemical cargoes, on C6 glioma cells occurs by crossing the BBB. They also revealed that the targeting of apoptosis-related genes in glioma-associated endothelial cells by ptc-miR396f resulted in a significant reduction in the gene expression of c-MYC. This led to the inhibition of tumor growth and an increase in the survival rate of mice with glioma. They also observed the downregulation of pro-tumoral cytokines, the induction of T cells, a suppression of regulatory T cells (Tregs) in TMEs, and an orchestrated activation of CAFs by TMEs [108].

Further, metastasis is a major cause of cancer morbidity and mortality, and it also refers to the dissemination of cancer cells from the primary tumor to neighboring tissues. Lan Yang et al. verified that ginseng-derived PDVLNs can prevent tumor cell epithelial–mesenchymal transition (EMT). Their research demonstrated that ginseng-derived PDVLNs exhibit significant inhibitory effects on the proliferation and migration of lung cancer-derived tumor cells (A549, NCI-H1299). Furthermore, treatment with ginseng-derived PDVLNs also resulted in reduced cell migration, invasion, clonal formation, and adhesion tube formation capabilities [110].

These series of studies have offered a promising strategy for tumor treatment, and they are, by themselves, enough to prove the suitability of PDVLN application in treating cancer.

Notably, beyond utilizing the intrinsic targeting capabilities of PDVLNs, there have been numerous endeavors to enhance the efficiency of their intracellular localization and to further change their targeting effects. Among these attempts, surface membrane engineering stands out as a prominent approach. This technique involves altering the exosome membrane by incorporating specific targeting ligands, including small molecules and genetic material, as well as facilitating cell membrane fusion. The most researchers have tried to attempt is to add folic acid (FA), as a ligand, to the surface of PDVLNs. One study reported that FA was displayed on the surface of ginger-derived PDVLNs in the targeted delivery of surviving siRNA to KB cancer models, which successfully inhibited the tumor growth on a xenograft model [35]. In another study, Niu W. et al., leveraged the ability of ginseng-derived PDVLNs to cross the blood–brain barrier by attaching doxorubicin-loaded heparin-based nanoparticles to them, thus achieving significant anti-glioma efficacy in glioma-bearing nude mice [111]. 

The remarkable targeting ability of PDVLNs reinforces their potential as a groundbreaking drug delivery platform as they are capable of overcoming the limitations of traditional drug delivery systems.

## 7. Conclusions and Future Directions

PDVLNs are cost-effective in production, as well as have non-toxic, non-immunogenic, and fascinating biocompatible properties, which enable PDVLNs to be a qualified drug delivery system. Additionally, their diverse sources and modifiable nature encourage further exploration of their potential in drug delivery. Despite these significant advantages, it is crucial to acknowledge some potential limitations in the current PDVLN research.

Our current understanding of PDVLN biogenesis, physiology, internalization, and cargo delivery remains limited, thus necessitating further investigations for elucidation; consequently, the risks associated with their clinical application remain elusive. 

The majority of animal experiments investigating the pharmacodynamics and efficacy of PDVLNs primarily focus on oral administration, with only a limited number opting for intravenous administration. However, no studies utilizing local injection or coating administration have been identified thus far. And it is imperative to explore alternative routes of delivery and compare their efficacy and toxicity in order to determine the optimal route for administering PDVLNs.

While the makers of mammalian exosomes are the transmembrane proteins CD9, CD81 [112], the markers of PDVLNs that can that can accurately reflect the localization, cellular origin, and secretion mechanism of PDVLNs remains insufficiently elucidated and warrants further investigation [113].

Although surface membrane engineering has made progress, there has not been any attempt in unloading the innate bioactive compound of PDVLNs, which should not compromise surface morphology, size, and biological functions.

Currently, the direct use of PDVLNs typically involves a simple screening and extraction process; thus, it lacks specialized drug design and optimization. Therefore, there is still room for improvement in areas such as tumor-targeting performance and the therapeutic efficacy of PDVLNs. To overcome these potential shortcomings, collaboration among medicinal chemists, pharmacists, and pharmacologists is essential to enhance the therapeutic effects of PDVLNs through detailed research and optimization.

In the ongoing research and optimization process, several aspects can be addressed. Firstly, optimizing the particle size and charge properties of PDVLNs can enhance their tumor-targeting capabilities. Secondly, detailed pharmacological studies will help to understand the mechanisms of action and therapeutic effects of PDVLNs in the human body. Additionally, the total synthesis of key pharmacological molecules and improvements in drug delivery systems are crucial steps to enhance the efficacy and safety of PDVLNs.

By systematically considering these factors and conducting comprehensive research and optimization, the application prospects of PDVLNs as drugs can be further expanded. Achieving the full potential of PDVLNs in clinical treatment will require interdisciplinary collaboration and sustained efforts.

Overall, the potential benefits of PDVLNs are undeniable. Over the past decade, our research on PDVLNs has increasingly substantiated its potential in biomedicine, thus warranting further investigation by scholars in related fields. Nevertheless, we are merely scratching the surface of PDVLNs at present, and it is inevitable that challenges will arise in the pursuit of PDVLN drug delivery. With continued breakthroughs in research, it is reasonable to anticipate that these barriers will be addressed, as is customary with any emerging field.

## Figures and Tables

**Table 1:** The size distribution of PDVLNs from various plant sources and different isolation methods.

Source	Part	Isolation Method	Size	Yield	Ref.
Strawberry	Juice	Ultracentrifugation	30 to 191 nm	18 ± 3 μg/250 mL of juice	[32]
Grape	Juice	Ultracentrifugation	500 to 600 nm	/	[19]
Tomato	Juice	Ultracentrifugation and SEC	110 ± 10 nm	26 ± 11 mg/kg of tomato, 2.6 × 10^15^ particles/kg of tomato	[33]
Grapefruit	Juice	Ultracentrifugation	~132 nm	~1.70 × 10^11^ particles per mL	[20]
Grapefruit	Juice	Ultracentrifugation	~102.4 nm	/	[34]
Ginger	Rhizome	Ultracentrifugation	70.09 ± 19.24 nm	/	[21]
Ginger	Rhizome	membrane filtration, differential, ultracentrifugation, and equilibrium density gradient ultracentrifugation	~124 nm	~5 × 10^3^ particles per mL	[35]
Lemon	Juice	Ultracentrifugation	/	600 μg of vesicles from 240 mL	[21]
Broccoli	Flower heads	Ultracentrifugation	146.7 ± 7.2 nm	7.87 × 10^12^ ± 2.18 × 10^12^ particles per mL	[23]
		Ultracentrifugation and SEC	174.3 ± 5.5 nm	9.62 × 10^10^ ± 9.81 × 10^9^ particles per mL	
Carrot	Juice	Ultracentrifugation and SEC	~150 nm	3.2 × 10^11^ particles/g of carrot	[24]
Apple	Pulp	Ultracentrifugation	80 to 500 nm	5.5 × 10^9^ particles/mL	[23]
Orange	Juice	Ultracentrifugation and density gradient fractionation using sucrose gradients	101.4 ± 5.5 nm	/	[26]
Arabidopsis	Calli	Ultracentrifugation	222.8 ± 36.5 nm	1.8 × 10^10^ particles/g FW (FW, fresh weight)	[17]
	Leaf apoplastic fluid		283.6 ± 58.3 nm	2.9 × 10^10^ particles/g FW (FW, fresh weight)	
Tea	Leaf	Ultracentrifugation and density gradient fractionation using sucrose gradients	~100 nm	/	[36]
*D. morbifera*	Sap	Ultracentrifugation	~100 nm	~1.5 mg protein/10 g of sap, 1.53 × 10^9^ particles/g (from leaf)	[37]

**Table 3:** Different administration routes and the therapeutic activity of PDVLNs.

Administration Route	Source	Therapeutic Activity	Ref.
Oral administration	Tea leaf	Anti-inflammation of ulcerative colitis	[36]
IV injection	ginger	Anti-cancer	[35]
Intranasal administration	Grapefruit	Anti-cancer of glioma in olfactory bulb	[34]
Transdermal delivery	Broccoli	/	[6]

## Data Availability

Not applicable.
